# Safety Assessment of Neoadjuvant Pertuzumab Combined with Trastuzumab in Nonmetastatic HER2-Positive Breast Cancer in Postmenopausal Elderly Women of South Asia

**DOI:** 10.1155/2018/6106041

**Published:** 2018-04-19

**Authors:** Nadia Hussain, Amira S. A. Said, Zainab Khan

**Affiliations:** ^1^Department of Pharmaceutical Sciences, College of Pharmacy, Al Ain University of Science and Technology, Al Ain, UAE; ^2^Department of Clinical Pharmacy, College of Pharmacy, Al Ain University of Science and Technology, Al Ain, UAE; ^3^Gynaecology Oncology Department, Punjab Care Hospital, Lahore, Pakistan

## Abstract

**Aim:**

To evaluate the safety issues and adverse effects of using TCHP regimen (docetaxel, carboplatin, trastuzumab, and pertuzumab) versus TCP regimen (docetaxel, carboplatin, and trastuzumab) in older postmenopausal women with nonmetastatic HER2-positive breast cancer. HER2 overexpressed in 20–25% of breast cancer signals an aggressive form of breast cancer and is treated with trastuzumab and pertuzumab.

**Methods:**

The patient record database was accessed to identify all postmenopausal women in the Punjab Care hospital who were above 65 years old, with stages 1–3 HER2-positive breast cancer and treated with neoadjuvant TCHP and neoadjuvant TCP from 2013 till 2016.

**Results:**

In TCH-P group and TCH group, mild fatigue (34% versus 36%) and diarrhea (48% versus 49%) were most common toxicities. Fever in TCH-P group and TCH group (12% versus 13%) was common. Anorexia affected 21% and 16% of patients receiving TCH and TCHP regimen, respectively. Febrile neutropenia was higher in TCH-P group 13% (3/23) versus 4.5% (1/22) in TCH group. Also 27.2% (6/22) of TCH-P group was hospitalized for treatment related toxicities versus 21.7% (5/23) of TCH group.

**Conclusion:**

Comparing neoadjuvant TCP and neoadjuvant TCH-P showed TCH-P regimen had an acceptable toxicity profile. Severe cardiac dysfunction was not observed. Using TCH-P regimen can be considered as relatively safe therapeutic option for elderly postmenopausal women with nonmetastatic HER2-positive breast cancer.

## 1. Introduction

Breast cancer not only is the most prevalent diagnosed malignancy in women but also is considered as the second leading cause of death among women cancers. Although several studies have shown similar survival benefits from adjuvant or neo adjuvant chemotherapy (NAC), the latter appeared to be the preferable treatment option [1]. Posttreatment NAC has been practically valued to downstage primary tumors in most women even in those with small tumor sizes, thus allowing for more breast preservation and helping in preventing tumor cells spread into the circulation.

Therapeutic options and treatment risks in breast cancer treatment for elderly women are highly challenging whereas the least invasive regimen is the most appreciated for old age. With a worldwide increasing elderly population, an increased number of breast cancer diagnosed older women are expected. In the United States alone, there are approximately three million breast cancer survivors who are aged above 65 years [1, 2]. Most new breast cancer diagnoses occur in women less than 65 years of age (58%) but most breast cancer deaths (60%) occur in women aged 65 years and older (37% in women aged 75 years and older). Despite the fact that more improved therapeutic options are available, breast cancer mortality rates were reduced by as much as 15.3% for women aged 50–64 years; however, older women have not had this level of success with only 7.4% reduction in the risk of breast cancer death [3]. Late diagnosis in elderly plus reluctance to give proper radiation or chemotherapy with more concerns for treatment toxicity and effects on quality of life in old age group led to greater treatment failures [4]. Although many studies show that elderly women in good health are able to tolerate state-of-the-art breast cancer treatments with similar overall survival (OS) benefits compared to younger women [5], these patients are underrepresented in clinical trials and persistently excluded. Literature now suffers from limited information on acute and long-term toxicity among older patients which can contribute to age bias on behalf of the clinician.

HER2 overexpression leads to adverse clinical scenarios such as high grade tumors, with increased invasiveness, metastasis, and mortality rates [7, 8]. HER2 gene encodes a tyrosine kinase receptor that mediates diverse functions in normal cells and is crucially involved in oncogenesis [9].

The advent of inhibitors of tyrosine kinase-associated cell cycle activation protein as HER2-targeted therapy has improved this clinical scenario and opened a new era of a more specific targeted cancer therapy.

Trastuzumab was the first drug approved since 1998 by the Food and Drug Administration [10] as a humanized monoclonal antibody to the HER2 extracellular domain. However, studies suggest that long-term exposure to trastuzumab may cause resistance in up to 15% patients relapsing within 12 months of treatment [11], probably due to resistance by promoting HER2 heterodimerization with HER3. Next generation HER2 therapies such as pertuzumab were developed and reported to target epitope heterodimerization on HER2 protein, thus blocking HER2-HER3 dimerization which makes it helpful to overcome trastuzumab resistance [12]. The combination of pertuzumab, trastuzumab, and docetaxel was approved in 2012 and 2013 to be used for HER2-positive metastatic [13] and early stage breast cancer, respectively [14].

This combination has achieved improved pathologic complete response (pCR) when compared to therapies involving trastuzumab and docetaxel. The pCR achieved is 39% versus 21%, respectively [13, 15]. In terms of survival rate, a higher pCR has been correlated to an increase in survival rate [6, 16].

Toxicity has been a legitimate concern and studies have indicated that the observed reduction in disease-free and breast cancer related mortality in older patients occurred at the risk of greater toxicity [17].

Indeed, introduction of trastuzumab and other anti-HER2-positive drugs have revolutionized treatment of HER2-positive breast cancer to turn it into a highly treatable disease with improved therapeutic results. However, still, the best combination of anti-HER2-positive drugs to achieve low toxicity over long treatment period requires more light to be shed on it.

One of the most concerning side effects associated with trastuzumab is left ventricular systolic dysfunction (LVSD). Studies have shown that while receiving trastuzumab treatment, 19% of patients experienced a significant decline in left ventricular ejection fraction (LVEF) and up to 4% of them developed severe heart failure (HF) [18, 19]. Older patients appear to be more prone to cardiac toxicity especially when trastuzumab is used [18, 20]. Studies have indicated that older women are at risk of congestive heart failure (CHF) with the risk increasing to 5.4% after the age of sixty. Expert consensus opinion issued by the National Comprehensive Cancer Network (NCCN) has concluded that using trastuzumab is appropriate for most fit elderly women [21].

Febrile neutropenia caused by chemotherapy is a frequent medical emergency associated with severe complications in the emergency department (ED) [22]. In Asian patients there appears to be an increased risk of developing febrile neutropenia when treated with pertuzumab. This is more common in cycles 1–3 [23].

Documented studies of the safety outcomes following HER2 directed therapy involving pertuzumab are an area of research that requires more light to be shed. Studies assessing the safety of this neoadjuvant TCH-P regimen ADD THIS (docetaxel, carboplatin, trastuzumab, and pertuzumab) versus the TCP regimen (docetaxel, carboplatin, and trastuzumab) in older postmenopausal women is somewhat limited and, in our judgment, data based on retrospective study would be of value especially to postmenopausal women with higher risks of toxicity.

## 2. Aim

The primary objective of this study was to retrospectively evaluate the safety issues and adverse effects of using the TCHP regimen (docetaxel, carboplatin, trastuzumab, and pertuzumab) versus the TCP regimen (docetaxel, carboplatin, and trastuzumab) in older postmenopausal women that had nonmetastatic HER2-positive breast cancer in a retrospective setting.

The secondary objectives in the present study were to focus on cardiac adverse effects and febrile neutropenia.

## 3. Ethics Statement

The study protocol was approved by the Ethics committee of Punjab Care hospital, Lahore, Pakistan (IP-4091). The study was performed in accordance with Good Clinical Practice standards and the ethical principles that have their origin in the Declaration of Helsinki.

## 4. Material and Methods

The cancer database of Punjab Care hospital was accessed to identify all postmenopausal women above the age of 65 that had stages 1–3 HER2-positive breast cancer treated at the hospital with neoadjuvant TCHP and neoadjuvant TCP from 2013 till 2016. Individual patient records were reviewed for information concerning treatment-received, dosage used, and side effects. Patient information was obtained including age at diagnosis, tumour size, clinical stage, hormone receptor status, number of lymph nodes involved, and the number of axillary lymph nodes removed during surgery.

Side effect reports from the patient records were classified according to the common terminology criteria for adverse effects (CTCAE), Version 4.0, June 2010, National Institutes of Health, National Cancer Institute [24]. CTCAE grade 1 were categorized as mild; CTCAE grades 2-3 side effects were classed as moderate and those that corresponded with CTCAE grade 4 toxicities were classified as severe.

The following treatment was administered to patients. Patients on the TCH-P regimen (TCH-P group) received trastuzumab (Herceptin®, 8 mg/kg IV infusion over 90 minutes on the first day of every 21-day cycle which was adjusted to 6 mg/kg over 60 minutes on Cycle 2 and then adjusted to 6 mg/kg IV over 30 minutes on Cycles 3 through 6); pertuzumab (Perjeta®, 840 mg IV infusion over 60 minutes on Day 1 of Cycle 1 (plus 60 min. postinfusion observation) and, then, 420 mg IV infusion over 30 minutes on Day 1 of Cycles 2 through 6); carboplatin (Carboplatin®, AUC6, IV infusion over 30 minutes on Day 1); and docetaxel (Taxotere®, 75 mg/m^2^ IV infusion over 60 minutes on day 1). All patients also received subcutaneous pegfilgrastim prophylactically on Day 2 of each cycle. Patients receiving the TCP regimen (TCP group) received similar dosage and cycles of trastuzumab, carboplatin, and docetaxel but did not receive pertuzumab. Duration of therapy was up to six months.

Following required surgery (lumpectomy or mastectomy), both patient groups (TCH-P and TCP) continued trastuzumab alone every three weeks for a total of 52 weeks of therapy.

For evaluation of the cardiac safety purposes, patient records were reviewed for reporting of cardiac issues. Patients who received either therapy had echocardiograms performed prior to treatment, at 4 and 8 months after treatment. In our institution, the lower limit for LVEF by ECHO is considered 55%. Both group TCH-P and TCP patients received trastuzumab which was continued in the adjuvant setting to complete one full year of therapy.

## 5. Statistical Analysis

Package for Social Science (SPSS) Version 21 (SPSS Inc., Chicago IL, USA) was used for statistical analysis. The two treatment groups were compared using paired *t*-test and the calculated *p* value was considered to be significant if it was ≤ 0.05.

## 6. Results

A total of 45 patients who met the inclusion criteria were included in the study. Twenty-two of them were those who received TCHP regimen and twenty-three received the TCH regimen. Demographic details of both patients groups were described in Table 1. All patients were older than 65 and have been postmenopausal for over 12 months. The median age in our study was 66.5 years with a median follow-up time of approximately 12 months, from the time of the first breast cancer diagnosis. All patients had undergone ECOG performance status of 0-1. The ECOG scale helps to indicate the patient's level of functioning.

In the group TCHP, 92% of the twenty-two patients received all six planned cycles. There were 8% of patients who had to reduce the number of cycles due to adverse effects. Approximately, 18% (4/22) of patients required a dose reduction. Specifically, 5.1% of all patients in study had both docetaxel and carboplatin reduced, of which docetaxel alone was dose-reduced in 2.7% patients.

Of the 23 patients who received neoadjuvant TCP, 90% received all six planned cycles but 10% of patients (3/22) required a dose reduction due to adverse effects. Both docetaxel and carboplatin had to be reduced for 8.6% (2/23) of the patients. No patient in this group required a reduction of docetaxel alone.

In both group TCH-P and group TCH, mild fatigue (34% versus 36%) and diarrhea (48% versus 49%) were the most commonly observed toxicities. Fever in group TCH-P and group TCH (12% versus 13%) was also common. In the group TCH-P, 8% of patients had moderate degree of nausea similar to the 7% observed in group TCH. Up to 4% of patients in the TCH group had severe myalgias compared to 2% in the group TCHP. Anorexia affected a total of 21% of patients receiving TCH and 16% of patients on the TCHP regimen.

**Figure 1:**
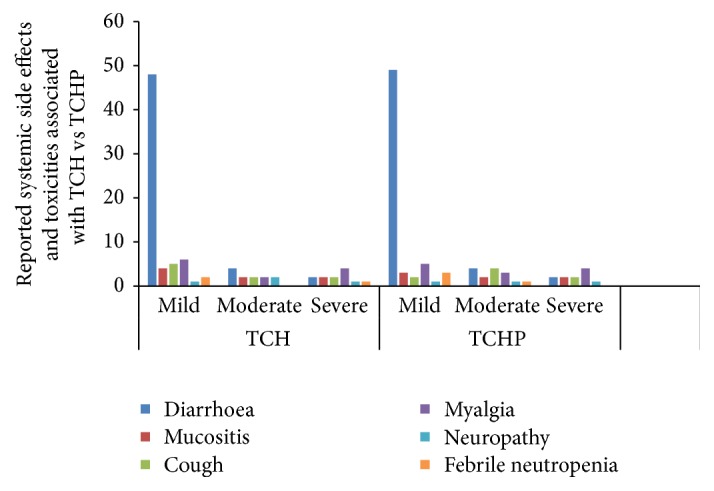
Reported systemic side effects and toxicities associated with TCH (docetaxel, carboplatin, and trastuzumab) versus TCHP (docetaxel, carboplatin, trastuzumab, and pertuzumab). The most common toxicities documented in the patient records by severity are shown. Numbers indicate percentages of patients with documented evidence of specific adverse toxicities. The severity of side effects was established according to Common Terminology Criteria for Adverse Events (CTCAE), Version 4.0, June 2010, National Institutes of Health, National Cancer Institute [6]. Mild side effects were classed as CTCAE grade 1, moderate as CTCAE grades 2-3, and severe as CTCAE grade 4 side effects.

**Figure 2:**
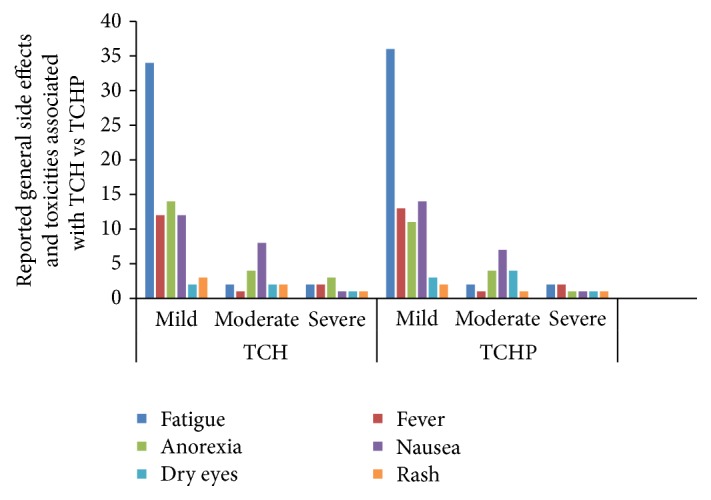
Reported general side effects and toxicities associated with TCH (docetaxel, carboplatin, and trastuzumab) versus TCHP (docetaxel, carboplatin, trastuzumab, and pertuzumab). The most common toxicities documented in the patient records by severity are shown. Numbers indicate percentages of patients with documented evidence of specific adverse toxicities. The severity of side effects was established according to Common Terminology Criteria for Adverse Events (CTCAE), Version 4.0, June 2010, National Institutes of Health, National Cancer Institute [6]. Mild side effects were classed as CTCAE grade 1, moderate as CTCAE grades 2-3, and severe as CTCAE grade 4 side effects.

In the group TCH, there were approximately 8% of patients who required treatment for febrile neutropenia; 1% of these were severe in nature which were aligned to CTCAE grade-4 toxicity. In the group TCHP, there were approximately 3 patients who required treatment for febrile neutropenia but none of these cases were severe. The rate of febrile neutropenia was higher in the TCH-P group 13% (3/23) versus 4.5% (1/22) in the TCH group. The most commonly reported toxicities associated with neoadjuvant TCH-P are detailed in Table 2. Also 27.2% (6/22) of the TCH-P group were hospitalized for treatment related toxicities versus 21.7% (5/23) of the TCH group.  Figure 1 shows the reported systemic side effects and toxicities associated with TCH (docetaxel, carboplatin, and trastuzumab) versus TCHP (docetaxel, carboplatin, trastuzumab, and pertuzumab).  Figure 2 shows the reported general side effects and toxicities associated with TCH (docetaxel, carboplatin, and trastuzumab) versus TCHP (docetaxel, carboplatin, trastuzumab, and pertuzumab).

To assess cardiac safety in all patients, pretreatment and posttreatment left ventricular ejection fractions were recorded for routine care. Patients who did not experience any cardiac symptoms had echocardiograms conducted every 4 months. Pretreatment LVEF in the group TCHP was 57% in 92% of patients. No patients experienced symptomatic left ventricular systolic dysfunction with TCH-P. Asymptomatic reductions in LVEF 10% were observed in only 2% of patients, which subsequently normalized in all patients. Pretreatment LVEF in the group TCH was 55% in 97% of patients and no patients experienced left ventricular systolic dysfunction with TCH-P. Only 1% of the patients in group TCH had asymptomatic reduction of 8% in LVEF.

## 7. Discussion

The objective of our retrospective analysis was to evaluate the safety profile of using TCH-P as neoadjuvant therapy in older postmenopausal women with nonmetastatic HER2-positive breast cancer. We compared this to older postmenopausal women who received TCH only.

Utilizing the TCH-P regimen has been associated with a higher pCR and an overall increased effectiveness in using dual HER2 blockade [13, 15]. However, trastuzumab and pertuzumab have side effects that can affect the patients' quality of life and ability to tolerate the treatment [25, 26].

In our patient population of both groups, fatigue (34% versus 36%) and diarrhea (48% versus 49%) were the most common side effects reported. Fever in group TCH-P and group TCH (12% versus 13%) was also common. However, febrile neutropenia was overall low compared to other studies [23, 27] and this could be due to the prophylactic use of pegfilgrastim on Day 2 as part of the standard care to the patients when the TCH-P regimen is used for them.

In group TCH-P, 8% of patients had moderate degree of nausea similar to the 7% observed in group TCH. Up to 4% of patients in the TCH group had severe myalgias compared to 2% in group TCHP. Anorexia affected a total of 21% of patients receiving TCH and 16% of patients on the TCHP regimen.

In group TCH, there were approximately 8% of patients who required treatment for cytopenias; 1% of these were severe in nature which were aligned to CTCAE grade 4 toxicity. In group TCHP, there were approximately 3% of patients who required treatment for cytopenias but none of these cases were severe.

Cardiac safety has been a genuine concern when treating patients with HER2 receptor blockers because blocking the HER2 signaling pathway can impair the normal stress response and cellular repair mechanisms of cardiomyocytes [28]. Baseline LVEF assessment before initiating treatment is important to evaluate existing heart disease that would be exacerbated by receiving potentially cardiotoxic anti-HER2 therapy. Follow-up cardiac monitoring during therapy allows for the detection of reduction in LVEF (even asymptomatic) but this requires careful consideration to avoid unnecessary halting of beneficial anti-HER2 therapy. The long-term consequences of asymptomatic LVEF decline in these patients is an area that requires further investigation. However, our results were consistent with that reported by the TRYPHAENA trial [27]. A recent study looking at the cardiac outcomes in patients receiving adjuvant weekly paclitaxel and trastuzumab for node-negative HER2-positive breast cancer also reported similar cardiac outcomes with paclitaxel and trastuzumab [29]. In the CLEOPATRA trial, the cardiac safety analysis part did not show an increase in the incidence of LVSD when dual anti-HER2 therapy was used [30]. Approximately 3.8% of patients receiving pertuzumab and trastuzumab had a significant decline in LVEF and this percentage was 6.6% in patients receiving trastuzumab. The incidence of symptomatic LVSD was low in the TCH group (1%) and no patient in the TCH-P group experienced symptomatic left ventricular systolic dysfunction. Asymptomatic reductions in LVEF 10% were observed in only 2% of patients, which subsequently normalized in all patients.

## 8. Conclusion

In conclusion, comparing neoadjuvant TCP and neoadjuvant TCH-P showed that the TCH-P regimen had an acceptable toxicity profile. Severe cardiac dysfunction was not observed, with no patients experiencing symptomatic or irreversible reductions in LVEF. This can be considered as a relatively safe therapeutic option for elderly postmenopausal women with nonmetastatic HER2-positive breast cancer.

## Figures and Tables

**Table 1:** Baseline clinical characteristics of the study patients (*n* = 45).

Demographic details	Group TCH (*n* = 23)	Group TCHP (*n* = 22)	*p* value
Age (yr)	68.3 ± 6.1	65.7 ± 7.1	0.69
Gender			
Females	22 (100%)	23 (100%)	0.95
Menopausal status at diagnosis	23 (100%)	22 (100%)	1.00
Clinical stage at diagnosis			
Stage I	1 (2.6)	2 (5.2)	0.99
Stage II	3 (7.8)	1 (2.6)	0.19
Stage III	01 (2.6)	02 (5.2)	0.55
Clinical nodal status			
Node positive	115 ± 21.9	122 ± 21.1	0.39
Node negative	80 ± 18.1	78 ± 19.8	0.91
Clinical T stage			
T1	3 ± 0.1	3 ± 0.1	1.00
T2	12 ± 0.3	12 ± 0.3	0.83
T3	6 ± 0.2	4 ± 0.2	0.88
T4	2 ± 0.1	1 ± 0.1	0.56
T4d	1 ± 0.1	1 ± 0.1	0.20
Pretreatment LEVF			
<55%	03 ± 2.1	05 ± 2.3	0.65
>55%	20 ± 5.3	17 ± 2.7	0.76
ECOG status			
0	9/23 (38%)	8/22 (34%)	1.00
1	9/23 (36%)	8/22 (33%)	1.00

**Table 2:** Reported side effects and toxicities associated with TCH versus TCHP. The numbers reflect percentages of patients with recorded side effects and toxicities in their electronic medical files. Mild, moderate, and severe side effects are correlated with CTCAE grade 1, grades 2-3, and grade 4 side effects, respectively.

Side effect/toxicity	TCH group (*n* = 23)				TCHP group (*n* = 22)			
Severity	Total (%)	Mild (%)	Moderate (%)	Severe (%)	Total (%)	Mild (%)	Moderate (%)	Severe (%)
*General side effects*								
Fatigue	36	34	2	2	40	36	2	2
Fever	14	12	1	2	16	13	1	2
Anorexia	21	14	4	3	16	11	4	1
Nausea	21	12	8	1	22	14	7	1
Dry eyes	5	2	2	1	8	3	4	1
*Gastrointestinal symptoms*								
Diarrhea	54	48	4	2	55	49	4	2
Mucositis	8	4	2	2	7	3	2	2
*Respiratory system side effects*								
Cough	9	5	2	2	8	2	4	2
*Dermatological side effects*								
Rash	6	3	2	1	4	2	1	1
*Musculoskeletal side effects*								
Myalgias	12	6	2	4	12	5	3	4
*Neurological side effects*								
Neuropathy	4	1	2	1	3	1	1	1
*Haematological side effects*								
Febrile neutropenia	13	2	0	1	4	3	1	0

## Data Availability

The datasets used and/or analyzed during the current study are available from the corresponding author upon reasonable request. All data generated or analyzed during this study are included in this article.

## Additional Points

*Limitations of the Study.* We are aware of certain limitations that the present study has. Our sample size is relatively small. Also due to the retrospective nature of our study, there is a potential of underreporting of adverse effects and toxicities associated with the TCH-P regimen.
